# The Association between Socioeconomic Factors and Visual Function among Patients with Age-Related Cataracts

**DOI:** 10.1155/2020/7236214

**Published:** 2020-11-30

**Authors:** Yu Wan, Yinhao Wang, Liming Zhao, Zhenyu Wang, Min Sun, Dongmei Chen, Yang Yang, Yanhui Xu, Shuxuan Lv, Yanan Yu, Xuemin Li, Aimin Jiang, Zhimin Chen

**Affiliations:** ^1^Department of Ophthalmology, Peking University Third Hospital, 49 North Garden Road, Haidian, Beijing 100191, China; ^2^Department of Ophthalmology, Beijing Fengtai Hospital, 1 Xi'an Street, Fengtai Town, Fengtai, Beijing 100071, China; ^3^Department of Ophthalmology, Huabei Petroleum General Hospital, Battle Road, Renqiu 062550, Hebei, China; ^4^Independent Researcher, Hebei, China; ^5^Department of Ophthalmology, The Hospital of Shunyi District Beijing, 3 Guangming South Street, Shunyi, Beijing 101300, China; ^6^Department of Ophthalmology, Hebei Eye Hospital, 399 Quanbeidong Street, Xingtai 054001, Hebei, China

## Abstract

**Background:**

With the development of the economy, socioeconomic factors, such as inequalities in the status of regional economies and the subsequent effects on health systems, have influenced the status of health. We explored the association between age-related cataracts and socioeconomic indicators, including the regional economy, health systems, and energy industries.

**Methods:**

This was a prospective, multicenter, Chinese population-based, cross-sectional study. A total of 830 participants from seven centers were enrolled. Data on the best-corrected visual acuity (BCVA), Lens Opacities Classification System III (LOCS III) score, Visual Function Index-14 (VF-14) score, total and subscale scores of the 25-item National Eye Institute Visual Functioning Questionnaire (NEI-VFQ-25), per capita disposable income (PCDI), medical resource-related indicators, and investments in the energy industry were obtained. Associations among these parameters were analyzed.

**Results:**

The PCDI ranking was correlated with the VF-14 score (*R* = −0.426, *P* < 0.01), total score of NEI-VFQ-25 (*r* = −0.500, *P* < 0.01), and BCVA (*r* = 0.278, *P* < 0.01). The number of health agencies (*r*1 = 0.267, *r*2 = −0.303, *r*3 = −0.291,), practicing or assistant practicing doctors (*r*1 = -0.283, *r*2 = 0.427, *r*3 = 0.502,), registered nurses (*r*1 = −0.289, *r*2 = 0.409, *r*3 = 0.469, *P* < 0.01), and health technicians (*r*1 = −0.278, *r*2 = 0.426, *r*3 = 0.500, *P* < 0.01) per 10,000 of the population was each correlated with the BCVA, VF-14 score, and total score of NEI-VFQ-25, respectively. Health expenditure per capita was correlated with the VF-14 score (*r* = 0.287, *P* < 0.01) and total score of NEI-VFQ-25 (*r* = 0.459, *P* < 0.01). The LOCS III P score was correlated with investments in the energy industry (*r* = 0.485, *P* < 0.001).

**Conclusions:**

Patients in higher economic regions with greater medical resources show a greater demand to undergo cataract surgery at a better subjective and objective visual function. The energy industry has a significant effect on cataracts, especially the posterior subcapsular cataract, and thus more attention should be paid to people in regions with abundant energy industries.

## 1. Introduction

Cataract is a major health issue that causes blindness or severe visual impairment [1]. There are different types of cataracts, including congenital cataracts, age-related cataracts, metabolic cataracts, and cataracts associated with certain syndromes, such as hereditary hyperferritinemia-cataract syndrome [2–4]. Cataract surgery can significantly improve the subjective and objective visual functions in patients [5–8]. However, not all patients with age-related cataracts are able or willing to have cataract surgery, especially in developing countries. This might depend on the cognitive levels of patients, their cultural differences, and socioeconomic factors. These differences are obvious between developed and developing countries. Developing countries are undergoing rapid economic transitions, and with rapid economic growth, industrialization, and urbanization, lifestyle and health behaviors tend to experience great changes [9], which in some way influence people's attitudes towards surgery.

In China, rapid growth in some regions has been accompanied by economic inequality [10, 11]. Besides, the regional economic status influences the regional health status [12], which may be caused by inequalities in the health systems [13]. For the choice of cataract surgery, this may also play an important role. Previous studies have reported that exposure to indoor air pollution from the burning of traditional biomass fuels (wood, charcoal, animal dung, and crop wastes) and coal constitutes a significant public health hazard, including cataract formation [14]. However, the effects on cataract formation caused by the energy industry, such as the mining and washing of coal and extraction of petroleum and natural gas, are seldom studied.

Therefore, we intend to explore the association between age-related cataracts and socioeconomic indicators, including the regional economy, health systems, and the energy industries, in order to provide a better understanding of their influence on cataract patients.

## 2. Methods

This was a prospective, cross-sectional, Chinese population-based, multicenter study. The research protocol was approved by the Peking University Third Hospital Ethics Committee following the tenets of the Declaration of Helsinki. Informed consent was obtained from the study participants.

### 2.1. Participant Selection

We prospectively collected the data of 830 participants (830 eyes from 830 participants) with age-related cataracts who underwent cataract surgery from March to June 2019 from seven centers in six districts: Haidian, Shunyi, Langfang, Cangzhou, Baoding, and Xingtai with Haidian and Shunyi from Beijing and the others from Hebei. Participants with age-related cataracts aged from 40 to 90 years were enrolled in this study. The exclusion criteria include the presence of glaucoma, fundus diseases, amblyopia, and history of ocular surgery and cognitive disorders.

### 2.2. Demographic and Clinical Data

Demographic and clinical characteristics, including age, sex, history of hypertension, diabetes, heart disease and cerebrovascular disease, best-corrected visual acuity (BCVA) of the operative eye (op-eye, ranging from 0 to 2.0 LogMAR), and the nuclear opalescence (NO), cortical (C), and posterior subcapsular (P) scores of the Lens Opacities Classification System III (LOCS III) were obtained.

The results of the Visual Function Index-14 (VF-14) and the National Eye Institute Visual Functioning Questionnaire (NEI-VFQ-25) were obtained from each subject before surgery. Every patient was asked to complete these two questionnaires by themselves. When there were problems with a patient's ability to read or understand the questionnaires, a single well-trained collector would help them read or explain the questions. Both questionnaires were translated into Chinese, and the scores were calculated based on a scoring algorithm. The NEF-VFQ-25 contained a total of 12 subscales: general health, general vision, ocular pain, near activities, distance activities, social functioning, mental health, role difficulties, dependency, driving, color vision, and peripheral vision. The higher the VF-14 or NEI-VFQ-25 score, the better the subjective visual function.

### 2.3. Socioeconomic Settings

In order to make the association more direct, we used a semiquantitative method. Using the per capita disposable income (PCDI) of the six districts from the local governments' Statistical Yearbooks in 2018, we ranked these districts from 1 to 6 (1 stands for the district with highest PCDI, 2 stands for the second, and so forth).

To assess the association of the healthcare services with visual function, we obtained the related data from the local governments' Statistical Yearbooks in 2018, including the number of health agencies, health technicians, practicing doctors (PDs) and assistant practicing doctors (APDs), registered nurses (RNs), people who had medical insurance, resident population, and public expenditure on medical and healthcare and family planning for the different regions. We define key indexes as follows:

① Health agencies per  10,000 population=the number of health agencies/resident population (ten thousand)
② PDs and APDs per  10,000 population=the number of PDs and APDs/resident population (ten thousand)
③ RNs per 10,000 population=the number of RNs/resident population (ten thousand)
④ Health technicians per 10,000population=the number of health technicians/resident population (ten thousand)
⑤ Health expenditure per capita=public expenditure on medical and health care and family planning/the number of people who had medical insurance

We divided the six districts into 3 groups, including relatively low, medium, and high investment according to the investment levels in the energy industry. The energy industry included the “mining and washing of coal,” “extraction of petroleum and natural gas,” and “processing of petroleum, coking, and processing of nuclear fuel.”

### 2.4. Data Analysis

IBM SPSS Statistics for Windows (Version 20.0. Armonk, NY : IBM Corp) and GraphPad Prism 5 for Windows (Version 5.01. GraphPad Software, Inc) were used for statistical analysis and graphs. All continuous variables and rank variables were expressed as the mean ± standard deviation (SD) and medians. Categorical variables were expressed as frequencies and percentages. The normality of each variable was tested using the one-sample Kolmogorov–Smirnov test. None of the variables were normally distributed. Therefore, the Mann–Whitney *U* test was used to compare the differences between the two independent samples. The Spearman coefficient correlation analysis was used to explore the relationship between two variables. A coefficient <0.4 indicated a weak correlation; ≥0.4∼0.7 indicated a moderate correlation; and ≥0.7 indicated a strong correlation. To adjust for confounding factors for BCVA, VF-14 score, and total score of NEI-VFQ-25, multiple linear regression was used. Baseline variables that were considered clinically relevant or that showed a univariate relationship with outcomes were entered into a multiple linear regression model. Finally, eleven confounding factors (including BCVA) were considered for VF-14 and NEI-VFQ-25 and ten confounding factors for BCVA. The level of significance was *P* < 0.05.

## 3. Results

A total of 830 eyes from 830 participants were included in this study. Demographic and clinical characteristics of the participants are presented in Table 1. The number of participants was 35 (4.2%), 75 (9.0%), 244 (29.4%), 334 (40.2%), and 142 (17.1%) for the different age groups 40–49, 50–59, 60–69, 70–79, and 80–90 years, respectively. The association of age with subjective and objective visual function measurements is presented in Supplementary Table S1 (in Supplementary Materials). Of the 830 participants, there were 339 (40.8%) male and 491 (59.2%) female patients. Males had higher total scores (mean 80.34 versus 75.95, *P* < 0.001) and subscale scores (data not shown) for the NEI-VFQ-25 than females. BCVA, LOCS III NO, C, P scores, and the VF-14 score showed no significant differences between males and females. The number of participants from the different districts was 348 (41.9%), 125 (15.1%), 181 (21.8%), 19 (2.3%), 25 (3.0%), and 132 (15.9%) from Haidian, Shunyi, Xingtai, Langfang, Baoding, and Cangzhou, respectively. There were 432 (52.0%) participants from Beijing and 398 (48.0%) subjects from Hebei.

**Figure 1:**
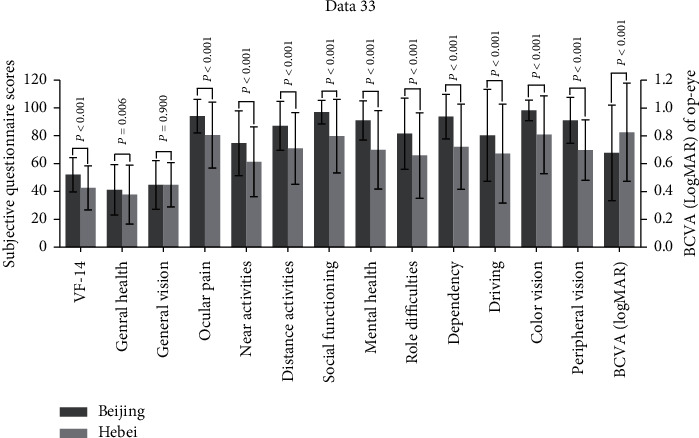
Difference in VF-14, NEI-VFQ-25, and BCVA of op-eye between Beijing and Hebei. VF-14, Visual Function Index-14; NEI-VFQ-25, 25-item National Eye Institute Visual Functioning Questionnaire; BCVA, best-corrected visual acuity. Error bar represents standard deviation.

**Figure 2:**
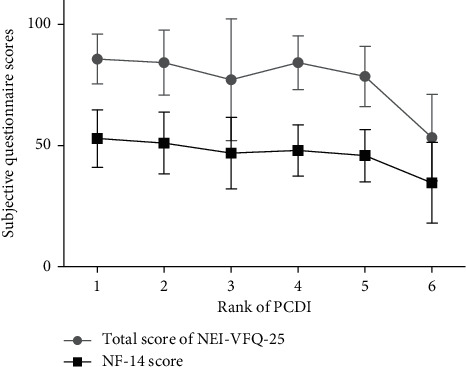
Change in subjective questionnaire scores with the rank of per capita disposable income (PCDI). VF-14, Visual Function Index-14; NEI-VFQ-25, 25-item National Eye Institute Visual Functioning Questionnaire.

A comparison of the VF-14 score, NEI-VFQ-25, and BCVA between Beijing and Hebei is shown in Figure 1. The PCDI of Beijing was higher than that of Hebei. The correlations of age and rank for PCDI of districts with BCVA, LOCS III scores, the VF-14 score, and total and subscale scores are presented in the Supplementary Table. With an increase in the PCDI rank, the VF-14 score and the total score of NEI-VFQ-25 tended to increase (Figure 2).

The association of the medical and healthcare services with subjective and objective measurements is presented in Table 2.

**Figure 3:**
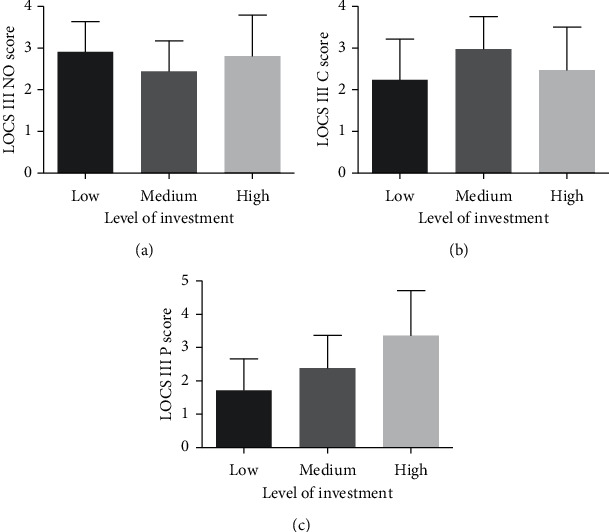
Bar graphs showing distributions of LOCS III scores among levels of investment in the energy industry. (a) Distribution of LOCS III NO score across levels of investment; (b) distribution of LOCS III C score across levels of investment; (c) distribution of LOCS III P score across levels of investment. VF-14, Visual Function Index-14; NEI-VFQ-25, 25-item National Eye Institute Visual Functioning Questionnaire; BCVA, best-corrected visual acuity; LOCS III, Lens Opacities Classification System III; NO, nuclear opalescence; C, cortical; P, posterior subcapsular.

The distributions of LOCS III NO, C, and P scores among the groups with energy industry investments are shown in Figure 3, and the results of the Kruskal–Wallis test indicated that the differences were statistically significant. The number of different levels of investments was 206 (24.8%), 276 (33.3%), and 348 (41.9%) for low, medium, and high levels, respectively. LOCS III NO (*r* = 0.158, *P* < 0.001) and C (*r* = 0.194, *P* < 0.001) scores were weakly correlated with the level of investment in “mining and washing of coal,” “extraction of petroleum and natural gas,” and “processing of petroleum, coking, and processing of nuclear fuel;” the LOCS III P score was moderately correlated with the level of investment (*r* = 0.485, *P* < 0.001).

This study used multiple linear regression to adjust for confounding factors. According to partial plots and scatter of studentized residuals with unstandardized predicted values, there was a linear relationship between independent variables and dependent variables, confirming that the data were equivariant and that observations were independent of one another (Durbin–Watson values were 1.843, 1.673, and 1.558 for BCVA, VF-14, and NEI-VFQ-25, respectively).

The tolerance was greater than 0.1, and there was no multicollinearity. Through the outlier test, a total of 15 BCVA outliers, three VF-14 outliers, and 13 NEI-VFQ-25 outliers were deleted according to the abnormal criteria that studentized deleted residuals >3 times of standard deviation for observations and leverage values >  0.2 or Cook's distance values  >  1. The regression models were statistically significant with the adjusted *R*^2^ = 0.20, 0.26, and 0.46 for BCVA, VF-14, and NEI-VFQ-25, respectively. The multiple linear regression model for BCVA, VF-14, and NEI-VFQ-25 is presented in Table 3.

## 4. Discussion

According to our data, participants who underwent cataract surgery aggregated in the age group of 60 to 79 years. Except for the LOCS III NO score, the VF-14 score, total score and certain subscale scores of NEI-VFQ-25, and age showed no correlation with other measurements. After adjusting for confounding factors, age was negatively associated with the VF-14 and the total score of NEI-VFQ-25. Age is thus a risk factor for cataracts [15]. In the multiple linear regression model, age was negatively correlated with the VF-14 score and the total score of NEI-VFQ-25, while it was not associated with the BCVA. Therefore, irrespective of the age of the occurrence of cataracts, the patients seemed to wait for the same level of problems with visual acuity before seeking care.

The phenomenon of younger participants with better subjective visual function undergoing cataract surgery was probably due to their higher job demand than the older participants. A participant's final decision for surgery relied more on their feelings about their visual function. In previous studies, the cataract blindness burden was higher for women, whereas men were more likely to receive cataract surgery [16–18]. We found a similar result that women accounted for approximately 60% of all the subjects, whereas their subjective visual function tended to be worse even after adjusting for confounding factors (1 for males and 2 for females during data entry). Previous studies have shown that women appeared to seek treatment at later stages of cataract formation [19] and were unwilling to pay for cataract surgery [20]. In China, more women are prone to depression or anxiety compared with men [12]. Both characteristics probably contributed to the worsening of their subjective visual function. Therefore, we need to pay more attention to complaints from women regarding their syndromes and visual function, rather than just focusing on their visual acuity.

Economic levels can have a variable influence on consultation and treatment rates for different diseases, including diabetes, inguinal hernia, gallstones, tonsillitis, varicose veins, cataracts, and osteoarthritis [21–23]. Previous studies have shown that there was an inverse U pattern between increasing deprivation and both patient consultation and operation ratios [21]. In our study, the distributions of subjective and objective visual function measurements varied among the different districts. The PCDI of each district showed a weak positive correlation with BCVA (LogMAR) and a moderate negative correlation with the VF-14 score and total and most subscale scores of the NEI-VFQ-25. Even after adjusting for confounding factors, such trends still existed for the BCVA, VF-14, and total score of NEI-VFQ-25. The PCDI is an important index that reflects the living standards of a population, and the BCVA, VF-14, or NEI-VFQ-25 reflected the visual function of patients. This suggested that people in relatively affluent regions have a greater demand for cataract surgery, even for less severe cataracts. Such a phenomenon may due to the demand for better visual function and quality of life among the population with development of the social economy. Therefore, the increase in the cataract surgery rate is probably due to the increasing demand for visual function and quality of life combined with the increasing incidence of cataracts, rather than solely for an increasing incidence of cataracts [24]. Cataracts are a huge economic burden [25], and cataract surgery is cost-effective and can probably promote economic development [26–29]. Therefore, a vicious cycle may exist where deprivation decreases the cataract surgery rate, and this low cataract surgery rate makes it hard for an individual or region to be lifted out of poverty.

The ability to pay can influence access to healthcare facilities in China [12, 30]. Low-income populations may be deprived of access to healthcare facilities and are less likely to have routine medical checkups. The inaccessibility to the healthcare system due to factors such as the uneven distribution of infrastructure, personnel and inadequate attention, and support from the government limits cataract surgeries [22, 31–33].

Health insurance has substantial effects on healthcare utilization (such as the use of physicians and preventive services) and health outcomes (such as self-reported health status and mortality conditional to injury and disease) [13]. Therefore, we collected health-related data and explored their association with subjective and objective visual function measurements of patients. Based on our study, in the regions with higher PDs, APDs, RNs, and health technicians per 10,000 population or a higher health expenditure per capita, patients with age-related cataracts decided to have cataract surgery at a higher score of the VF-14 and NEI-VFQ-25. This result was consistent with that of the previous studies, which indicated that the accessibility to the health system and health insurance promoted the implementation of cataract surgery. Knowledge of diagnosis and treatment was also one of the factors influencing the decision-making for cataract surgery [22], and the low density of health-related personnel in the regions of patients with low VF-14 scores and total scores of NEI-VFQ-25 indirectly supported that idea. A low density of health-related personnel means fewer opportunities to obtain health-related consultation and knowledge. However, the results for health agencies were inconsistent, suggesting a potentially low utilization of the health agency [34].

Household air pollution from burning of solid fuels for cooking, including coal and biomass fuels (wood, crop residues, and dung), has caused many health problems, such as an increase in disability-adjusted life years and reduced life expectancy [35]. The form of energy used was proven to be associated with high levels of indoor air pollution and an increase in the incidence of cataracts either in adults or children [14, 36]. Some studies reported that the use of kerosene was associated with nuclear and posterior subcapsular cataracts, especially among women who are normally responsible for food preparation and cooking [37, 38]. In our study, we found that the severity of posterior subcapsular cataracts increased with an increase in the level of investment in the energy industry. This phenomenon may share a mechanism with indoor energy use. Therefore, people in areas where the energy industry accounts for the majority should pay more attention to posterior subcapsular lens opacity. People in such areas should actively seek regular medical examination and inform doctors in their region regarding their exposure to coal and petrol. Doctors should be sensitive to a patients' native place and focus on changes to the posterior subcapsular cataracts. Furthermore, people paying attention to their visual function should be encouraged to visit an eye doctor when they feel their visual function is worsening because the LOCS III P score was associated with objective as well as subjective visual function after adjusting for confounding factors. However, the association in our study was indirect, and the different types of energy use were not analyzed. Further research on this aspect is recommended.

## 5. Conclusion

In conclusion, women tend to have a lower self-assessment of their visual function when receiving cataract surgery. Patients in regions at a higher economic level with greater medical resources show a greater demand to undergo cataract surgery at a better subjective and objective visual function. The energy industry has a significant effect on cataracts, especially posterior subcapsular cataracts. Therefore, more attention should be paid to people in regions with an abundant energy industry. With the development of the economy, cataract surgery can transform from a surgery to prevent blindness to surgery for a better visual function-related quality of life.

## Figures and Tables

**Table 1:** Demographic and clinical characteristics, VF-14 score, and NEF-VFQ-25 scores of the participants (*n* = 830).

Variables	Mean (SD)	Median
Age (year)	77.75 (18.64)	84.85
BCVA (LogMAR)	0.75 (0.36)	0.70
LOCS III NO score	2.78 (0.80)	3.00
LOCS III C score	2.46 (0.99)	3.00
LOCS III P score	2.19 (1.22)	2.00
VF-14	47.52 (14.82)	45.83
NEF-VFQ-25		
General health	39.49 (19.73)	50.00
General vision	44.72 (16.75)	40.00
Ocular pain	87.64 (19.79)	100.00
Near activities	68.30 (25.04)	75.00
Distance activities	79.38 (23.40)	87.50
Social functioning	88.77 (21.06)	100.00
Mental health	80.93 (24.41)	93.75
Role difficulties	74.00 (29.19)	87.50
Dependency	83.39 (26.39)	100.00
Driving (*n* = 283)	74.16 (34.73)	91.67
Color vision (*n* = 821)	89.92 (21.87)	100.00
Peripheral vision	82.20 (24.31)	100.00
Total score	77.75 (18.64)	84.85

BCVA, best-corrected visual acuity; NO, nuclear opalescence; C, cortical; P, posterior subcapsular; LOCS III, Lens Opacities Classification System III; VF-14, Visual Function Index-14; NEI-VFQ-25, 25-item National Eye Institute Visual Functioning Questionnaire.

**Table 2:** Correlations of the medical and healthcare services with subjective and objective measurements (*n* = 830).

	Number of health agencies per 10,000 population	Number of PDs and APDs per 10,000 population	Number of RNs per 10,000 population	Number of HTs per 10,000 population	Health expenditure per capita (yuan)
BCVA (LogMAR)	0.267^*∗∗*^	−0.283^*∗∗*^	−0.289^*∗∗*^	−0.278^*∗∗*^	−0.020
LOCS III NO score	−0.174^*∗∗*^	0.239^*∗∗*^	0.236^*∗∗*^	0.237^*∗∗*^	0.159^*∗∗*^
LOCS III C score	0.223^*∗∗*^	−0.298^*∗∗*^	−0.294^*∗∗*^	−0.296^*∗∗*^	−0.164^*∗∗*^
LOCS III P score	0.487^*∗∗*^	−0.368^*∗∗*^	−0.393^*∗∗*^	−0.368^*∗∗*^	0.063
VF-14	−0.303^*∗∗*^	0.427^*∗∗*^	0.409^*∗∗*^	0.426^*∗∗*^	0.287^*∗∗*^
General health	−0.130^*∗∗*^	0.156^*∗∗*^	0.150^*∗∗*^	0.155^*∗∗*^	0.153^*∗∗*^
General vision	−0.012	−0.017	−0.012	−0.019	0.107^*∗∗*^
Ocular pain	−0.229^*∗∗*^	0.434^*∗∗*^	0.406^*∗∗*^	0.432^*∗∗*^	0.471^*∗∗*^
Near activities	−0.259^*∗∗*^	0.396^*∗∗*^	0.368^*∗∗*^	0.399^*∗∗*^	0.267^*∗∗*^
Distant activities	−0.265^*∗∗*^	0.452^*∗∗*^	0.430^*∗∗*^	0.448^*∗∗*^	0.414^*∗∗*^
Social functioning	−0.314^*∗∗*^	0.538^*∗∗*^	0.510^*∗∗*^	0.533^*∗∗*^	0.502^*∗∗*^
Mental health	−0.289^*∗∗*^	0.539^*∗∗*^	0.504^*∗∗*^	0.537^*∗∗*^	0.367^*∗∗*^
Role difficulties	−0.197^*∗∗*^	0.379^*∗∗*^	0.351^*∗∗*^	0.379^*∗∗*^	0.418^*∗∗*^
Dependency	−0.358^*∗∗*^	0.589^*∗∗*^	0.559^*∗∗*^	0.586^*∗∗*^	0.400^*∗∗*^
Driving	−0.064	0.331^*∗∗*^	0.275^*∗∗*^	0.337^*∗∗*^	0.358^*∗∗*^
Color vision	−0.336^*∗∗*^	0.564^*∗∗*^	0.540^*∗∗*^	0.558^*∗∗*^	0.496^*∗∗*^
Peripheral vision	−0.315^*∗∗*^	0.508^*∗∗*^	0.484^*∗∗*^	0.504^*∗∗*^	0.427^*∗∗*^
Total score of NEI-VFQ-25	−0.291^*∗∗*^	0.502^*∗∗*^	0.469^*∗∗*^	0.500^*∗∗*^	0.459^*∗∗*^

^*∗*^ *P* < 0.05, ^*∗∗*^*P* < 0.01, PDs, practicing doctors; APDs, assistant practicing doctors; RNs, registered nurse; HT, health technician; BCVA, best-corrected visual acuity; NO, nuclear opalescence; C, cortical; P, posterior subcapsular; LOCS III, Lens Opacities Classification System III; VF-14, Visual Function Index-14; NEI-VFQ-25, 25-item National Eye Institute Visual Functioning Questionnaire.

**Table 3:** Multiple linear regression model for BCVA, VF-14, and NEI-VFQ-25.

Variables	BCVA			VF-14			NEI-VFQ-25		
	Β	SE	SC	*β*	SE	SC	*β*	SE	SC
(Constant)	0.116	0.089	—	71.028	3.780	—	108.525	3.834	—
Age	−0.001	0.001	−0.017	−0.166	0.048	−0.112^*∗∗*^	−0.156	0.049	−0.088^*∗∗*^
Gender	−0.010	0.021	−0.015	−1.127	0.897	−0.038	−3.508	0.911	−0.100^*∗∗*^
BCVA	—	—	—	−5.603	1.369	−0.137^*∗∗*^	−9.201	1.398	−0.188^*∗∗*^
LOCS III NO score	0.124	0.014	0.301^*∗∗*^	0.947	0.623	0.052	0.960	0.634	0.045
LOCS III C score	0.061	0.011	0.184^*∗∗*^	-0.846	0.474	−0.058	−0.579	0.484	−0.033
LOCS III P score	0.024	0.009	0.087^*∗∗*^	1.049	0.384	0.088^*∗∗*^	2.663	0.392	0.188^*∗∗*^
Hypertension	−0.031	0.023	−0.046	2.030	0.965	0.069^*∗*^	2.837	0.982	0.081^*∗∗*^
Diabetes	0.024	0.026	0.030	−1.453	1.107	−0.042	−0.889	1.129	0.022
Heart disease	0.033	0.032	0.033	−1.380	1.366	−0.032	−1.684	1.386	−0.033
Cerebrovascular disease	0.027	0.054	0.016	−0.331	2.258	−0.005	−1.762	2.283	−0.021
Rank of PCDI	0.044	0.006	0.267^*∗∗*^	−3.157	0.259	−0.435^*∗∗*^	−5.135	0.264	−0.597^*∗∗*^

^*∗*^ *P* < 0.05, ^*∗∗*^*P* < 0.01, SE, standardized error; SC, standardized coefficients; NO, nuclear opalescence; C, cortical; P, posterior subcapsular; LOCS III, Lens Opacities Classification System III; VF-14, Visual Function Index-14; NEI-VFQ-25, 25-item National Eye Institute Visual Functioning Questionnaire; PCDI, per capita disposable income.

## Data Availability

The datasets used and analyzed during the current study are available from the corresponding author on reasonable request.

## Supplementary Materials

Supplementary Materials Table S1: correlations of age and rank of PCDI with BCVA, LOCS III scores, VF-14, and NEI-VFQ-25 (*n* = 830).
