# Supplementary material for: The Association between Socioeconomic Factors and Visual Function among Patients with Age-Related Cataracts
Source: J Ophthalmol. 2020 Nov 30;2020:7236214. doi: 10.1155/2020/7236214 (PMC7722637; doi:10.1155/2020/7236214)
Supplement: Supplementary Materials — Table S1: correlations of age and rank of PCDI with BCVA, LOCS III scores, VF-14, and NEI-VFQ-25 (n = 830). [file 7236214.f1.docx]

**Supplementary Table. Correlations of age and rank of PCDI with BCVA, LOCS III scores, VF-14 and NEI-VFQ-25 (n=830)**

|  | Age | Rank of PCDI |
| --- | --- | --- |
| BCVA (LogMAR) | 0.044 | 0.278** |
| LOCS III NO score | 0.307** | -0.237** |
| LOCS III C score | 0.049 | 0.296** |
| LOCS III P score | -0.038 | 0.368** |
| VF-14 | -0.071* | -0.426** |
| General Health | -0.179** | -0.155** |
| General Vision | -0.077* | 0.019 |
| Ocular Pain | 0.065 | -0.432** |
| Near Activities | -0.125** | -0.399** |
| Distance Activities | -0.101** | -0.448** |
| Social Functioning | 0.015 | -0.533** |
| Mental Health | 0.033 | -0.537** |
| Role Difficulties | -0.029 | -0.379** |
| Dependency | -0.054 | -0.586** |
| Driving | -0.011 | -0.337** |
| Color Vision | 0.002 | -0.558** |
| Peripheral Vision | -0.063 | -0.504** |
| Total score of NEI-VFQ-25 | -0.072* | -0.500** |

* P < 0.05

** P < 0.01

NO, nuclear opalescence; C, cortical; P, posterior subcapsular; LOCS III, Lens Opacities Classification System III; VF-14, Visual Function Index-14; NEI-VFQ-25, 25-item National Eye Institute Visual Functioning Questionnaire; PCDI, per capita disposable income
